# The Association of Dietary Diabetes Risk Reduction Score and the Risk of Cancer: A Systematic Review and Meta-Analysis of Observational Studies

**DOI:** 10.3390/nu17233802

**Published:** 2025-12-04

**Authors:** Zahra Maghsoudi, Saleh A. Alsanie, Yohannes Adama Melaku, Aliakbar Sayyari, Mehran Nouri, Marzieh Shoja, Beheshteh Olang, Habib Yarizadeh, Behzad Zamani

**Affiliations:** 1 Iranian Social Security Organization, Tehran 14579-65595, Iran; 2 Department of Basic Health Sciences, College of Applied Medical Sciences, Qassim University, Buraydah 51452, Saudi Arabia; 3 Department of Clinical Nutrition, Medical City, Qassim University, Buraydah 51432, Saudi Arabia; 4 Flinders Health and Medical Research Institute, College of Medicine and Public Health, Flinders University, Adelaide, SA 5042, Australia; 5 Cancer Epidemiology Division, Cancer Council Victoria, Melbourne, VIC 3004, Australia; 6 Pediatric Gastroenterology, Hepatology and Nutrition Research Center, Research Institute for Children’s Health, Shahid Beheshti University of Medical Sciences, Tehran 15514-15468, Iran; 7 Infertility and Reproductive Health Research Center, Health Research Institute, Babol University of Medical Sciences, Babol P.O. Box 47135-547, Iran; 8 Nutrition and Metabolic Diseases Research Center, Clinical Sciences Research Institute, Ahvaz Jundishapur University of Medical Sciences, Ahvaz 61357-15794, Iran; 9 Children Emergency Department, Karolinska University Hospital, 17176 Stockholm, Sweden; 10 Social Determinants of Health Research Center, Saveh University of Medical Sciences, Saveh P.O. Box 3919676651, Iran

**Keywords:** diabetes risk reduction diet, DRRD, cancer, meta-analysis

## Abstract

Background/Objectives: Several studies have suggested a contrasting link between a diabetes risk reduction diet (DRRD) pattern and cancer risk; however, their findings have been inconsistent. This study aims to systematically review observational studies and, where possible, quantify the overall effect through a meta-analysis. Methods: Searches were conducted in PubMed, Scopus, and Web of Science through May 2025. Odds ratios (ORs), along with their confidence intervals, were extracted for meta-analysis. The random-effects model was used to combine the ORs. Results: Nineteen studies met the inclusion criteria for the systematic review and meta-analysis. Of these, six reports examined the relationship between the DRRD and breast cancer risk, three assessed liver cancer incidence, two analyzed pancreatic cancer risk, and two focused on endometrial cancer. Additionally, seven studies explored the association with other cancers, including ovarian, colorectal, renal, head and neck, bladder, and lung cancers. The meta-analysis revealed that high adherence to the DRRD is associated with a decreased cancer risk (OR = 0.77, 95% confidence interval [95% CI]: 0.71–0.84, *p* < 0.001). Conclusions: After stratifying by geographic region, gender, study design, and cancer site, the inverse relationship remained significant across all subgroups. DRRD can be viewed as a beneficial approach associated with a lower cancer risk.

## 1. Introduction

Cancer, also referred to as malignant tumors or neoplasms, encompasses a wide range of diseases that can arise in nearly any part of the body [1]. It is generally defined as an abnormal and uncontrolled proliferation of cells that can invade nearby tissues and potentially spread to other organs [1]. While breast, lung, colorectal, prostate, skin, and stomach cancers have the highest prevalence, respectively, the deadliest cancers are lung, colon and rectum, liver, stomach, and breast ones [2]. The global mortality rate associated with cancer reached approximately 10 million people in 2020, making it one of the leading causes of death [2].

Cancer has become a significant public health concern. The burden of cancer is not limited to the physical and emotional effects on patients; it also places financial strain on communities and healthcare systems [2,3]. It is estimated that global cancer expenditures will reach USD25.2 trillion between 2020 and 2050 [3]. This evidence highlights the need for countermeasures to prevent and treat cancers. Current research shows that 30 to 50% of cancer cases can be prevented by reducing exposure to known risk factors and adopting evidence-based preventive measures [2].

A variety of risk factors, including obesity, diabetes, poor nutrition, a sedentary lifestyle, and various environmental influences, may contribute to the development of cancer. Epidemiological studies have suggested a relationship between diabetes and certain cancers [4]. Age, obesity, smoking, physical inactivity, and unhealthy diets are shared risk factors for diabetes and cancer [4,5]. A healthy and balanced diet is essential for preventing and managing both diabetes and cancer [6].

The connection between nutrition and cancer risk has been explored, demonstrating that while some foods increase cancer risk [7], others have protective effects [8]. Nonetheless, emphasizing overall dietary patterns instead of individual nutrients or foods can serve as a stronger predictor due to their potential interactions [9]. Different dietary patterns have been identified as preventive or causative factors for non-communicable diseases [10]. The Diabetes Risk Reduction Diet (DRRD) is an innovative dietary pattern for diabetes prevention. It emphasizes the consumption of more grain fiber, nuts, coffee, polyunsaturated fats, and whole fruits while minimizing the intake of saturated and trans fats, red and processed meats, sugary beverages, and foods with a high glycemic index [11].

Due to the overlapping mechanisms of diabetes and cancer, which primarily involve hyperinsulinemia, insulin resistance, and pro-inflammatory effects [12,13], it has been proposed that DRRD may also have beneficial effects on cancer prevention. Several studies have evaluated the connection between DRRD and cancer risk, yet the results remain inconclusive. While many of them discovered an inverse association [14,15,16], some did not find a significant relationship [17,18]. This study sought to elucidate the relationship between the dietary diabetes risk reduction score and cancer risk via a systematic review and meta-analysis.

## 2. Materials and Methods

The present study was conducted following the reporting standards outlined by the Preferred Reporting Items for Systematic Reviews and Meta-Analyses (PRISMA) guidelines. We registered this systematic review and meta-analysis in the Open Science Framework (OSF) database; registration number: 10.17605/OSF.IO/TPCRS.

### 2.1. Search Strategy

A thorough search of the electronic databases PubMed/Medline, SCOPUS, and ISI Web of Science was conducted to gather relevant articles published until May 2025. The following keywords were used together for this purpose: “Dietary diabetes risk reduction score” OR “DDRRS” OR “Diabetes Risk Reduction Diet” OR “DRRD” OR “Type 2 Diabetes Prevention Diet” AND “neoplasms” OR “Neoplasm” OR “cancer” OR “carcinoma” OR “neoplastic disease” OR “malignant*” OR “cancer”(Table S1). Additionally, the reference lists of existing studies were manually searched to identify any potential articles that could meet the inclusion criteria. No language restrictions were applied. If the full text of an article was not available, we contacted the corresponding author via email.

### 2.2. Literature Inclusion and Exclusion Criteria

The studies that met the following PECOS criteria were eligible for inclusion in the meta-analysis:

1. [P] Population: Adults aged ≥18 years without baseline cancer.
2. [E] Exposure: Dietary diabetes risk reduction score (DDRRS).
3. [C] Comparison: The highest vs. the lowest DDRRS.
4. [O] Outcomes: Estimated cancer risk (all cancers, e.g., colorectal, breast, liver, etc.).
5. [S] Study design: Observational studies, including case–control and cohort studies.

The data with the following characteristics were excluded: (1) non-observational research, such as trials, reviews, letters, congress papers, magazines, and gray literature; (2) studies with non-human subjects; (3) specific populations such as pregnant or breastfeeding women, children, teenagers, and individuals with critical medical conditions.

### 2.3. Data Screening and Extraction

First, all identified articles from the databases were imported into EndNote. After removing duplicates, two authors (MS and MN) screened the articles based on their titles and abstracts. Next, the full texts of the remaining publications were reviewed to select eligible articles according to the inclusion criteria. Any discrepancies were resolved through discussions between the relevant authors.

Following the selection step, two authors (ZM and HY) extracted and tabulated the necessary data from the included studies. Data were cross-checked by the authors, and controversies were resolved by a third author (BZ). The extracted information was as follows: the first author’s name, country, publication year, study design, characteristics of participants (gender and age range or mean age), sample size, cancer site, effect sizes (relative risk [RR], odds ratio [OR], and hazard ratio [HR]) with 95% confidence intervals (CI), comparison groups (tertile, quartile, and quantile), and adjusted variables.

### 2.4. Assessment of Study Quality

The Newcastle–Ottawa Scale (NOS) was used to evaluate the quality of the included studies. This tool is specifically designed for assessing observational studies, including case–control and cohort studies. The NOS score ranges from 0 to 9, and a study is deemed high-quality if it has a score of 7 or higher.

### 2.5. Statistical Analysis

In this study, all statistical analyses were performed using Stata software version 14 (Stata Corp LP, College Station, TX, USA). The meta-analysis involved pooling the multivariable adjusted RRs, HRs, or ORs from the highest versus the lowest categories of DDRRS according to a random effects model (due to significant heterogeneity among studies), applying the DerSimonian-Laird method. Values from each study and their corresponding standard errors were converted to their natural logarithms to stabilize variances and normalize their distributions. To visually display the ORs and 95% CIs of the included studies, a forest plot was used.

The Cochrane Q test and the I^2^ statistic were used to assess the heterogeneity among the studies. A *p*-value less than 0.1 for the Q test and an I^2^ value above 50% were considered significant. Studies were grouped by gender, cancer site, and study design for subgroup analysis to identify potential sources of heterogeneity. Both visual inspection of the funnel plot and Egger’s test, with a threshold *p*-value below 0.05, were used to evaluate publication bias. Additionally, a sensitivity analysis was conducted to determine each study’s impact on the overall effect size.

## 3. Results

### 3.1. Studies Selection

**Figure 1:**
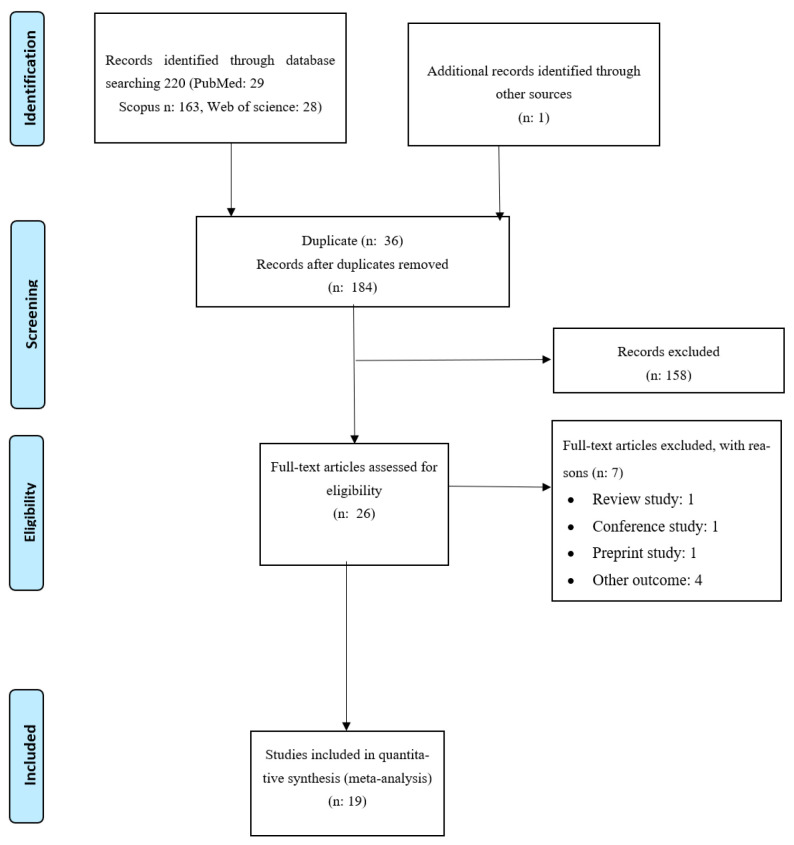
PRISMA flow diagram for study selection.

The process of identifying eligible studies is illustrated as a flowchart in Figure 1. A total of 220 citations were recorded through database searches (PubMed: 29, Scopus: 163, Web of Science: 28). After removing duplicates, 184 publications were screened based on their titles and abstracts. Finally, 26 full-text articles were reviewed, of which 19 evaluated the relationship between the diabetes risk-reduction diet score and cancer risk.

### 3.2. Description of Eligible Studies

The main characteristics of the included studies are summarized in Table 1. All the retrieved studies were published between 2019 and 2025 [14,15,16,17,18,19,20,21,22,23,24,25,26,27,28,29,30,31,32], including eight case–control [14,16,17,18,21,23,24,27] and eleven cohort studies [15,19,20,22,25,26,28,29,30,31,32]. Eight of the included studies originated from the USA, of which five were from the PLCO (Prostate, Lung, Colorectal, and Ovarian Cancer Screening Trial) [22,25,26,28,29], one from the NHS (Nurses’ Health Study) and HPFS (Health Professionals Follow-up Study) [19], one from NHS and NHSII [20], one from WHI (Women’s Health Initiative) [15]. Another was extracted from EPIC (European Prospective Investigation into Cancer and Nutrition) [31] and one from China (cohorts of the Shanghai Men’s Health Study (SMHS) and the Shanghai Women’s Health Study (SWHS)) [32]. In addition, three studies originated from Iran [17,18,23], five were conducted in Italy [14,16,21,24,27], and one was conducted in Spain (SUN project) [30].

Overall, six studies investigated the association between DRRDS and the risk of breast cancer [17,18,20,23,24,30]. Three articles addressed liver cancer [15,19,32], two focused on the pancreatic [14,22], one on colorectal [16], two on the endometrial [21,31], one on ovarian [27], one on the lung [25], one on renal [29], one on the bladder [26], one on the head and neck [28]. The nine studies included participants of both genders [14,16,19,22,25,26,28,29], while ten others focused solely on females [15,17,18,20,21,23,24,27,30]. The studies had a quality score ranging from 7 to 9, indicating they were of high quality.

### 3.3. Meta-Analysis

#### Overall Analysis

**Figure 2:**
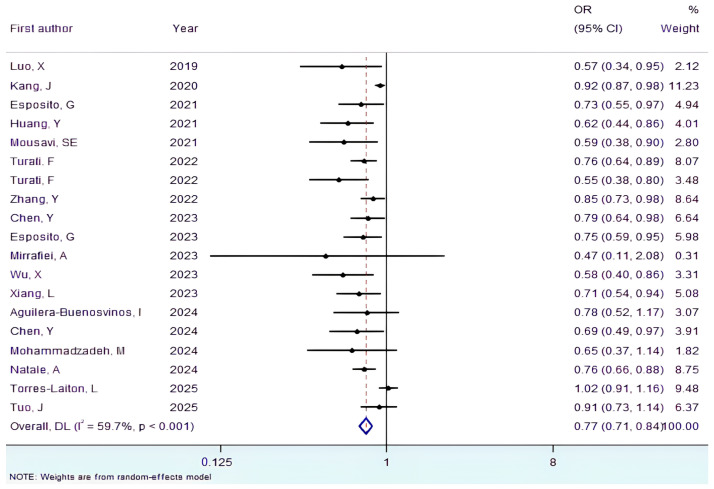
Forest plot illustrating the relationship between the diabetes risk-reduction diet score and cancer risk [14,15,16,17,18,19,20,21,22,23,24,25,26,27,28,29,30,31,32]. In addition to the overall analysis, subanalyses for cohort and case–control studies are included.

Overall, 19 studies were included in the meta-analysis, comprising 11 cohort studies and eight case–control studies. As shown in Figure 2, pooled analysis indicated that higher adherence to the diabetes risk reduction diet score was associated with a 23% lower risk of developing any cancer compared with lower adherence (OR = 0.77, 95% CI: 0.71–0.84, *p* < 0.001, I^2^ = 59.7%, P_-Heterogeneity_ < 0.001). Although this inverse association was also observed within the subset of cohort studies (OR = 0.82, 95% CI: 0.74–0.90, *p* < 0.001, I^2^ = 59.6%, P_-Heterogeneity_ = 0.006) and case–control studies (OR = 0.73, 95% CI: 0.67–0.80, *p* < 0.001, I^2^ = 0.0%, P_-Heterogeneity_ = 0.751), it was found to be more significant in case–control studies group. As five of the included studies originated from the PLCO study cohort, whose population may overlap, and their effect estimates may be correlated, the assumption of independence may be violated. To address this problem, the results were first combined using a random-effects model. In another model, we pooled the effect sizes of the five PLCO studies (Figure S1) using a fixed-effects model (OR = 0.77, 95% CI: 0.70–0.85, *p* < 0.001, I^2^ = 30.4%, P-_Heterogeneity_ = 0.219). Then we combined these into a single effect size using the results from other studies. The pooling of these data showed that studies with a shared population did not materially affect the overall finding (OR = 0.79, 95% CI: 0.72–0.86, *p* < 0.001, I^2^ = 64.5%, P_-Heterogeneity_ < 0.001).

### 3.4. Subgroup Analysis

Because of the heterogeneity observed, a series of predefined analyses were carried out to pinpoint its source and to further examine the link between DRRDS and cancer risk (see Table 2). As noted earlier, when studies were grouped by design, the inverse association between DRRDS and cancer risk remained significant in both case–control and cohort studies. Although there was notable heterogeneity among cohort studies (I^2^ = 59.6%, *p* = 0.006), this heterogeneity was not present in case–control studies (I^2^ = 0.0%, *p* = 0.751). Among the included studies, ten recruited only female subjects, while nine included both sexes and reported overall cancer risk as well as gender-specific stratified results. Therefore, in our subgroup analysis by gender, we treated the data for each gender from these studies as separate entries. The pooled OR for females was OR = 0.84 (95% CI: 0.78–0.91), and for males was OR = 0.72 (95% CI: 0.63–0.81). No substantial heterogeneity was observed for the male subgroup (I^2^ = 30.8%, *p* = 0.172), whereas the female subgroup showed heterogeneity (I^2^ = 38.6%, *p* = 0.045).

By geographic region, the pooled OR of cancer for DRRDS was 0.76 (95% CI: 0.67, 0.87) in U.S. studies, 0.79 (95% CI: 0.67, 0.90) in European studies, and 0.77 (95% CI: 0.71, 0.84) in Asian studies. Significant heterogeneity was observed among U.S. (I^2^ = 62.8%, *p* = 0.009) and European studies (I^2^ = 69.8%, *p* = 0.003), but not in Asian studies (I^2^ = 29.0%, *p* = 0.238). Eventually, the stratifying based on cancer site showed that adherence to DRRDS is inversely associated with all subgroups of female cancers (0.82, 95% CI: 0.74, 0.92), gastrointestinal cancers (0.72, 95% CI: 0.62, 0.83), and others, including renal, bladder, lung, and head and neck (0.78, 95% CI: 0.68, 0.88). A noticeable heterogeneity was seen in the female cancers subgroup (I^2^ = 58.3%, *p* = 0.014), whereas no heterogeneity was observed in the gastrointestinal (I^2^ = 38.3%, *p* = 0.151) and other (I^2^ = 23.7%, *p* = 0.269) subgroups.

### 3.5. Publication Bias and Sensitivity Analysis

**Figure 3:**
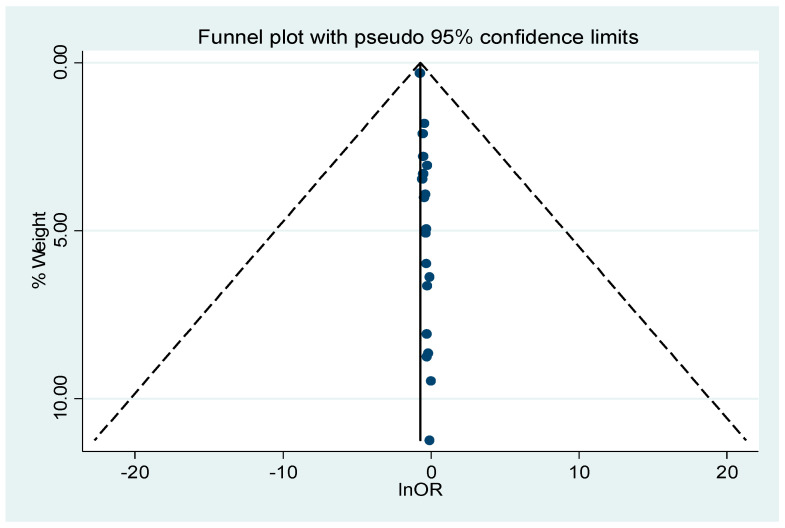
The funnel plot of the included studies.

Publication bias was evaluated using Egger’s regression test and inspection of the funnel plot. The analysis revealed significant evidence of bias (*p* = 0.001), which was further supported by the asymmetry observed in the funnel plot (Figure 3).

The effect of a single study on the pooled OR was explored by sensitivity analysis. The results of this test indicated that removing any of the included studies did not substantially change the overall effect size (Figure S3).

## 4. Discussion

To our knowledge, this is the first systematic review and meta-analysis examining the link between a diabetes risk reduction diet and cancer risk. Our key findings indicate that high adherence to the DRRD has a reverse association with cancer risk compared to low adherence. The overall findings remained consistent across subgroups defined by geographic region, cancer site, sex, and study design.

Several dietary patterns have been used to assess the association between diet and cancer risk, including the Mediterranean diet, DASH diet, and Dietary Inflammatory Index (DII) [33,34,35]. Overall, these dietary patterns provide a broader understanding of dietary intake than individual nutrients or foods when evaluating the risk of nutrition-related diseases. The DRRD is a relatively novel dietary pattern created based on components associated with type 2 diabetes [11]. An inverse association between a high DRRD score and disorders such as obesity, diabetes, and cardiovascular diseases has been observed in diverse populations [11,36,37].

Shared mechanisms, such as genetic predispositions, metabolic dysregulation (including hyperinsulinemia and hyperglycemia), chronic inflammation, oxidative stress, and lifestyle factors, suggest that preventive approaches for type 2 diabetes might also benefit cancer [38,39]. The DRRD is a healthy dietary pattern distinguished from other well-defined patterns by its inclusion of key dietary components linked to the risk of type 2 diabetes [11]. In a U.S. population survey, following the DRRD was linked to lower all-cause, cardiovascular, and cancer mortality [40]. The results of this meta-analysis show a protective association between DRRD and cancer risk; however, this association is stronger among men than among women. These findings were also observed in several studies with different cancers [14,19,22]. This may be because women’s estrogen offers protection [41], reducing the impact of the diet. Also, insulin resistance is more significant in men than in women [42]. Therefore, the effects of DRRD, which target insulin resistance, are more pronounced in males. In addition, the results in the case–control subgroup were more notable, which could be due to recall bias or selection bias, potentially exaggerating effect estimates [43].

Although the overall analysis indicates a negative relationship of DRRD to cancer risk, some inconsistencies exist among the included studies, as some did not find a significant association [17,18]. Aguilera-Buenosvinos et al. concluded that a moderate, not high, adherence to the Dietary-Based Diabetes Risk Score is associated with lower breast cancer risk [30], which underscores the anti-carcinogenic effects of this antidiabetic diet. This conflicting finding with the DRRD score may arise from the fact that the Dietary-Based Diabetes Risk Score assesses overall dietary fiber rather than just fiber from cereals, considers PUFA intake instead of the PUFA: SFA ratio, and encompasses groups such as vegetables, fruits, low-fat dairy, and whole grain cereals, which are only part of the Dietary-Based Diabetes Risk Score.

Many studies have examined how components of the DRRD score relate to cancer risk. Cao et al., in a dose–response meta-analysis, demonstrated that higher nut intake is correlated with lower risks of both cancer incidence and overall mortality. Specifically, an incremental consumption of 10 g of tree nuts per day was linked to a 20% decrease in cancer-related deaths [44]. In addition, the results of an umbrella review of meta-analyses indicated that coffee consumption reduced the incidence of several cancer sites; however, it was directly associated with urothelial carcinoma and bladder cancer [45]. On the other hand, a comprehensive systematic review and meta-analysis of 148 publications concluded that consuming red and processed meat increases the likelihood of various cancers [46]. Moreover, when the association of dietary glycemic index with twenty-three cancer sites was evaluated, a positive relationship was reported; however, the certainty of the evidence was low [47].

Several putative mechanisms have been proposed to explain the results, primarily emphasizing the insulin-sensitizing properties of DRRD. Insulin-like growth factor-1 (IGF-1) has demonstrated mitogenic and antiapoptotic effects and is responsible for stimulating the growth and proliferation of all cell types [48]. IGF-1 provides cells with abundant nutrients, allowing them to undergo hypertrophy and cell division [49]. Several studies have indicated that elevated IGF-1 levels may be linked to a higher risk of cancer and cancer mortality [50,51]. IGF-1 receptors are widely distributed throughout the body and possess a high affinity for insulin [52]. So, hyperinsulinemia or insulin resistance may lead to an increased production of IGF-1 [12,53], which, in turn, contributes to cancer promotion by stimulating cell proliferation [51,54]. Moreover, insulin was shown to induce the migration ability of cancer cells, contributing to metastasis [55]. In a cohort study, Tsujimoto et al. showed that cancer mortality was significantly higher in those with hyperinsulinemia [56]. Insulin resistance was proven to increase the odds of cancer mortality independently of diabetes [57]. Chronic hyperinsulinemia was also shown to induce tumor growth in breast and pancreatic cancers [58,59]. Metformin, widely prescribed as a first-line treatment for type 2 diabetes, has been linked in multiple studies to lower mortality rates among women with breast cancer [60,61]. Therefore, dietary modifications aimed at reducing insulin resistance, such as adhering to the DRRD, could be a beneficial approach for cancer prevention.

Moreover, a higher DRRD score indicates a higher intake of coffee, nuts, cereal fiber, and fruits, which ensures an increased consumption of antioxidants, dietary fiber, and polyphenols [62,63,64]. These elements have been strongly shown to promote cancer prevention [48,49,50] by neutralizing reactive ROS and mitigating oxidative stress [65]. The DRRD also features a low intake of trans and saturated fatty acids, sugar-sweetened beverages, as well as red and processed meats. Consumption of processed foods may elevate cancer risk due to high levels of nitrates and nitrites, which can form carcinogenic compounds like nitrosamines, known to be linked to certain cancers [66]. Additionally, processed foods contain advanced glycation end products (AGEs), which have been shown to promote inflammation and oxidative stress [67], both of which contribute to cancer development [68]. Trans fats can induce inflammation by increasing oxidative stress, altering gene expression, impairing insulin sensitivity, and disrupting gut microbiota [69]. Sugar-sweetened beverages may also contribute to cancer development by causing gut microbiome dysbiosis and triggering the release of pro-inflammatory mediators [70]. On the other hand, some components of DRRD, such as cereal fiber, whole fruits, and nuts [71,72], have prebiotic effects that help balance the gut microbiome and, in turn, lower the risk of cancer [71]. Furthermore, adherence to DRRD could promote weight loss, which is beneficial since it can nullify the effects of fat-induced carcinogens [72]. Nonetheless, it remains unclear whether any of the mentioned mechanisms individually contribute to the anticancer effects of DRRD or whether they act synergistically. In addition, it is important to note that these mechanisms were not directly evaluated in the included studies and should be regarded as plausible, hypothesis-generating explanations. Therefore, future research that includes biomarker measurements will be crucial to validate these pathways and enhance causal inference.

This is the first study to investigate the association between DRRD and cancer risk comprehensively. However, some limitations should be noted. Most dietary assessment tools rely on a predefined food list, which may not fully capture participants’ eating habits. Unmeasured food items could introduce confounding factors, resulting in biased estimates of the link between DRRD and cancer. Additionally, because self-reported food intake can be prone to measurement error and reporting bias, the DRRD scores may not accurately reflect actual adherence to the diet. Five of the included studies from the PLCO study shared a population that violated the assumption of independent data; however, we attempted to address this issue by pooling them and treating them as a single dataset, while also conducting subgroup analyses based on cancer site. Furthermore, our analysis showed moderate to high heterogeneity across studies, indicating caution when generalizing these findings. Finally, our analysis revealed significant publication bias, which could threaten the validity of the conclusions drawn from the meta-analysis.

## 5. Conclusions

In conclusion, this study identified a significant inverse relationship between following the DRRD and overall cancer risk, consistent across subgroup analyses by geographic region, cancer type, gender, and study design. However, given the high heterogeneity and evidence of publication bias, careful interpretation is essential to confirm these findings. Further research using a prospective design along with biomarker measurement and clinical trial intervention is necessary to establish causality and elucidate the biological pathways behind the observed associations.

## Figures and Tables

**Table 1:** General characteristics of included studies.

Number	First Author (Year)	Country	Study Design	Cancer Site	Sex	Sample Size	Age Range or Mean Age (y)	Dietary Assessment	Effects	Comparison	Adjustments	Quality Score
1	Luo, X. (2019) [19]	USA	Cohort -NHS and HPFS	Liver	Both	Control: 137,608 Case: 160	W: 30–55 M: 40–75	FFQ	HR	Q4 vs. Q1	Age, study period, gender, race, physical activity, smoking status, body mass index, aspirin use, alcohol intake, total calorie intake, and type 2 diabetes	8
2	Kang, J (2020) [20]	USA	Cohort-NHS and NHSII	Breast	Female	Control: 182,654 Case: 11,943	25–55	FFQ	HR	Q5 vs. Q1	race, family income; age at menarche, age at menopause, postmenopausal hormone use, oral contraceptive use history, parity, age at first birth, breastfeeding history, family history of breast cancer, history of benign breast disease, height, alcohol intake, total caloric intake, total vegetable intake, physical activity, and BMI at age 18 y, change in weight since age 18 y	7
3	Esposito, G. (2021) [21]	Italy	Case-Cntrol	Endometrial	Female	Control: 908 Case: 454	18–79	FFQ	OR	High vs. medium-low	age, education, year of interview, BMI, physical activity, smoking, alcohol intake, history of diabetes, total energy intake, age at menarche, parity, menopausal status, use of oral contraceptives, and use of hormone replacement therapy	8
4	Huang, Y. (2021) [22]	USA	Cohort -PLCO	Pancreatic	Both	Control: 101,729 Case: 402	65.5	FFQ	HR	Q4 vs. Q1	Age, sex, smoking status, former smoking, alcohol consumption, physical activity, BMI, aspirin use, history of diabetes, family history of pancreatic cancer, and energy intake	7
5	Mousavi, SE. (2021) [23]	Iran	Case–Control	Breast	Female	Controls: 700 Cases: 350	62.5	FFQ	OR	Q4 vs. Q1	Age, energy intake, education, residency, family history of breast cancer, physical activity, marital status, smoking, alcohol consumption, supplement use, breastfeeding, menopausal status, and BMI	7
6	Turati, F. (2022) [24]	Italy	Case–Control	Breast	Female	Controls: 2588 Cases: 2569	23–74	FFQ	OR	Q4 vs. Q1	Study center, age, education, year of interview, BMI, physical activity, smoking, history of diabetes, parity, menopausal status, age at menopause, use of oral contraceptives, hormone replacement therapy, family history of breast cancer, alcohol intake, and total energy intake.	7
7	Turati, F. (2022) [14]	Italy	Case–Control	Pancreatic	Both	Controls: 652 Cases: 326	34–80	FFQ	OR	T3 vs. T1	Age, sex, year of interview, education, BMI, tobacco smoking, history of diabetes, alcohol intake, and total energy intake	8
8	Zhang, Y. (2022) [25]	USA	Cohort -PLCO	Lung	Both	Control: 98,159 Case: 1632	65.5	FFQ	HR	Q4 vs. Q1	Age, sex, BMI, energy intake, family history of lung cancer, marital status, race/ethnicity, smoking status, alcohol intake, history of diabetes	7
9	Chen, Y. (2023) [26]	USA	Cohort -PLCO	Bladder	Both	Control: 99,001 Case: 761	62.41	FFQ	HR	Q4 vs. Q1	Age, sex, BMI, energy intake, family cancer history, marital status, race, cigarette smoking status, alcohol intake status, and diabetes history	7
10	Esposito, G. (2023) [27]	Italy	Case–Control	Ovarian	Female	Controls: 2411 Cases: 1031	17–79	FFQ	OR	Q4 vs. Q1	Age, center, year of interview, education, total energy intake, history of diabetes, menopausal status, parity, use of oral contraceptives, family history of ovarian/breast cancer	7
11	Mirrafiei, A. (2023) [18]	Iran	Case–Control	Breast	Female	Controls: 150 Cases: 149	24–73	FFQ	OR	Q5 vs. Q1	Age, energy intake, education, marital status, menopause status, alcohol use, smoking, use of vitamin supplements, medicines, medical history (diabetes, hypertension, and hyperlipidemia), history of hormone and oral contraceptive use, age at first menarche (year), time since menopause in postmenopausal women, weight at age 18 years, number of children, physical activity level, length of breastfeeding, family history of breast cancer, and body mass index	8
12	Wu, X. (2023) [28]	USA	Cohort -PLCO	Head and neck	Both	Control: 101,755 Case: 279	65.53	FFQ	HR	Q vs. Q1	Age, sex, race, marital status, educational level, BMI, smoking status, pack-years of smoking, drinking status, alcohol consumption, history of diabetes, family history of HNC, and energy from diet	7
13	Xiang, L. (2023) [29]	USA	Cohort -PLCO	Renal	Both	Control: 101,755 Cases: 446	65.5	FFQ	HR	Q4 vs. Q1	Age, sex, race, marital status, educational level, body mass index, smoking status, smoking pack-years, alcohol consumption, ibuprofen use, arm (intervention or control), family history of renal cancer, history of diabetes, history of hypertension, and energy intake from diet	7
14	Aguilera-Buenosvinos, I. (2024) [30]	Spain	Cohort-SUN	Breast	Female	Control: 10,810 Case: 147	35	FFQ	HR	T3 vs. T1	Age, height, years at university, family history of breast cancer, smoking status, lifetime tobacco exposure, physical activity, TV watching, alcohol intake, BMI, age of menarche, age at menopause, history of pregnancy, months of breastfeeding, use of hormone replacement therapy, energy intake, prevalence of diabetes, family history of diabetes, and use of oral contraceptives	9
15	Chen, Y. (2024) [15]	USA	Cohort-WHI	Liver	Female	Control: 98,786 Case: 216	50–79	FFQ	HR	T3 vs. T1	Age, energy intake, study group indicator (observational study, clinical trial), race/ethnicity, education, smoking status, alcohol consumption, physical activity, body mass index, nonsteroidal anti-inflammatory drugs use, oral contraceptive use, menopausal hormone therapy use, family history of cancer, self-reported liver disease, and self-reported diabetes	9
16	Mohammadzadeh, M. (2024) [17]	Iran	Case–Control	Breast	Female	Controls: 267 Cases: 134	>30	FFQ	OR	T3 vs. T1	Age, BMI, physical activity, energy intake, smoking, first pregnancy age, cancer family history, marital status, education, and menopausal status	7
17	Natale, A. (2024) [16]	Italy	Case–Control	Colorectal	Both	Controls: 4154 Cases:1953	19–74	FFQ	OR	T3 vs. T1	sex, age, study center, education, occupational physical activity, body mass index, alcohol consumption, tobacco smoking, family history of intestinal cancer, aspirin use, menopausal status and postmenopausal hormone use, and total energy intake.	8
18	Torres-Laiton, L (2025) [31]	Europe	Cohort -EPIC	Endometrial	Female	Control: 285,418 Case: 1955	50.13	FFQ	HR	T3 vs. T1	Age at recruitment and country, and adjusted by menopausal status, smoking status, ever use of hormone treatment for menopause and BMI	9
19	Tuo, J (2025) [32]	China	Cohort -SMHS and SWHS	Liver	Both	Control: 132,524 Case: 687	40–74	FFQ	HR	T3 vs. T1	Calorie intake, BMI, physical activity, education, smoking status, family history of liver cancer, medical history of chronic hepatitis and T2DM, and alcohol drinking status	8

Abbreviations: FFQ: food frequency questionnaire; HR: hazard ratio; OR: odds ratio; BMI: body mass index.

**Table 2:** Results of the meta-analyses examining the association between the diabetes risk reduction diet and cancer risk; subgroup analyses by study design, gender, geographic region, and cancer site are presented.

Groups	*n*	Highest vs. Lowest Comparison				
		OR (95% CI)	*p*-Value	I^2^	P_Heterogeneity_	P_Between-group_
Analysis on overall cancers						
Overall analysis	19	0.77 (0.71–0.84)	<0.001	59.7%	<0.001	
Subgroup by study design						
Case–control studies	8	0.73 (0.67–0.80)	<0.001	0.0%	0.751	0.008
Cohort studies	11	0.82 (0.74–0.90)	<0.001	59.6%	0.006	
Subgroup by gender						
Female	19	0.84 (0.78–0.91)	<0.001	38.6%	0.045	0.009
Male	9	0.72 (0.63–0.81)	<0.001	30.8%	0.172	
Subgroup by geography region						
USA	8	0.76 (0.67–0.87)	<0.001	62.8%	0.009	0.730
Europe	7	0.79 (0.67–0.90)	0.001	69.8%	0.003	
Asia	4	0.75 (0.57–0.98)	0.035	29.0%	0.238	
Subgroup by cancer site						
Female cacers	9	0.82 (0.74–0.92)	0.001	58.3%	0.014	0.057
Gastrointestinal cancers	6	0.72 (0.62–0.83)	<0.001	38.3%	0.151	
Others	4	0.78 (0.68–0.88)	<0.001	23.7%	0.269	

## Supplementary Materials

The following supporting information can be downloaded at: https://www.mdpi.com/article/10.3390/nu17233802/s1, Figure S1: Pooled analysis of PLCO studies; Figure S2: Pooled effect size of PLCO as a single study in combination with other studies; Figure S3: The sensitivity analysis of included studies; Table S1: Search Syntax.
