# Supplementary material for: The Association of Dietary Diabetes Risk Reduction Score and the Risk of Cancer: A Systematic Review and Meta-Analysis of Observational Studies
Source: Nutrients. 2025 Dec 4;17(23):3802. doi: 10.3390/nu17233802 (PMC12693901; doi:10.3390/nu17233802)
Supplement: Supplementary file 1 [file nutrients-17-03802-s001.zip › nutrients-3938850-supplementary.pdf]

Supplementary Table S1. Search Syntax

PubMed	("Dietary diabetes risk reduction score"[All Fields] OR "DDRRS"[All Fields] OR "Diabetes Risk Reduction Diet"[All Fields] OR "DRRD"[All Fields]) OR "Type 2 Diabetes Prevention Diet"[All Fields]) AND ("neoplasms"[MeSH Terms] OR ("neoplasm s"[All Fields] OR "neoplasms"[MeSH Terms] OR "neoplasms"[All Fields] OR "neoplasm"[All Fields]) OR "cancer*"[All Fields] OR ("carcinoma"[MeSH Terms] OR "carcinoma"[All Fields] OR "carcinomas"[All Fields] OR "carcinoma s"[All Fields]) OR "neoplastic disease"[All Fields] OR "malignan*"[All Fields])	29
Scopus	((ALL ("dietary diabetes risk reduction score") OR ALL ("DDRRS") OR ALL ("diabetes risk reduction diet") OR ALL ("DRRD") OR ALL ("type 2 diabetes prevention diet"))) AND ((ALL ("malignan*") OR ALL ("carcinoma*") OR ALL ("cancer*") OR ALL ("neoplasm*")))	163

Web of Science ISI	(((((ALL=("Dietary diabetes risk reduction score"))) OR ALL=(DDRRS)) OR ALL=("Diabetes Risk Reduction Diet")) OR ALL=(DRRD)) OR ALL=("Type 2 Diabetes Prevention Diet") AND ((((ALL=("neoplasm*")) OR ALL=("cancer*")) OR ALL=("carcinoma*")) OR ALL=("malignan*"))	28

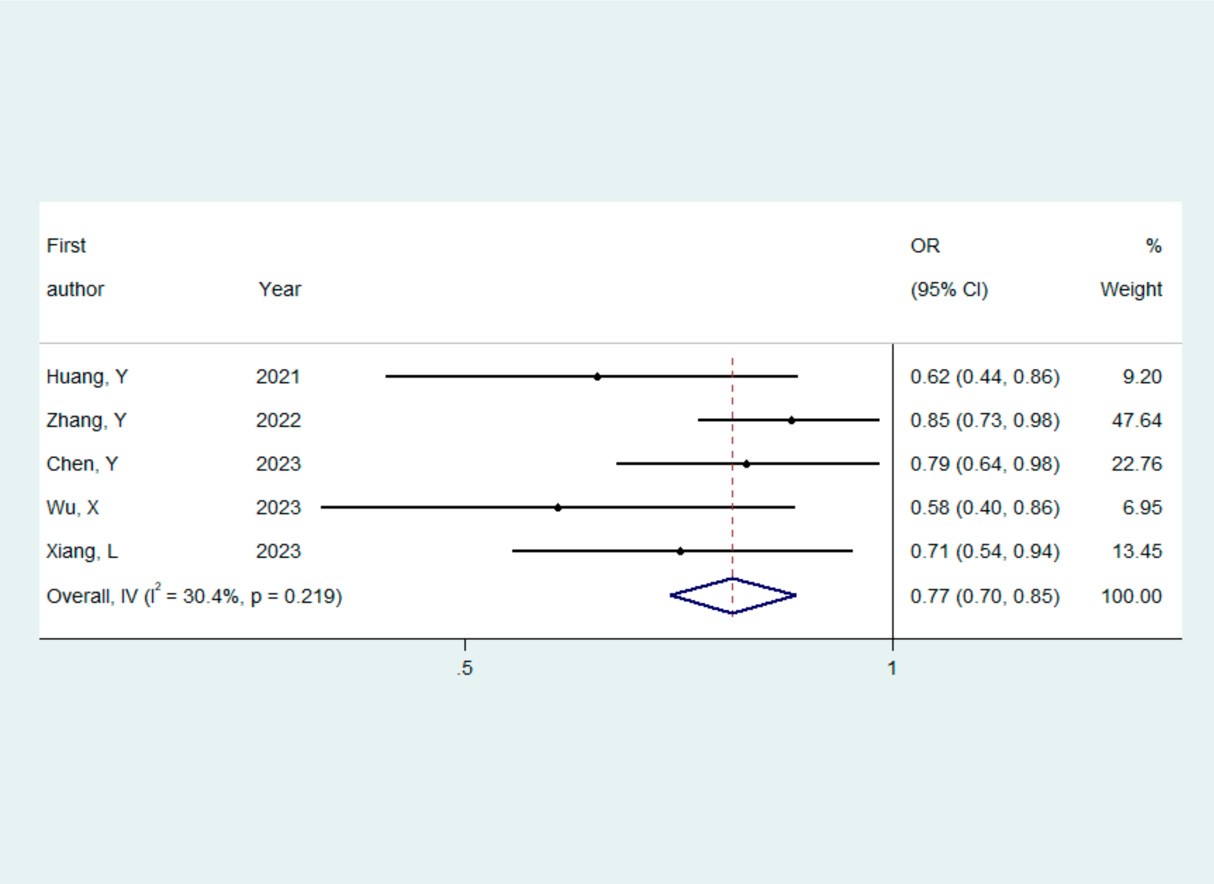

Figure S1. Pooled analysis of PLCO studies

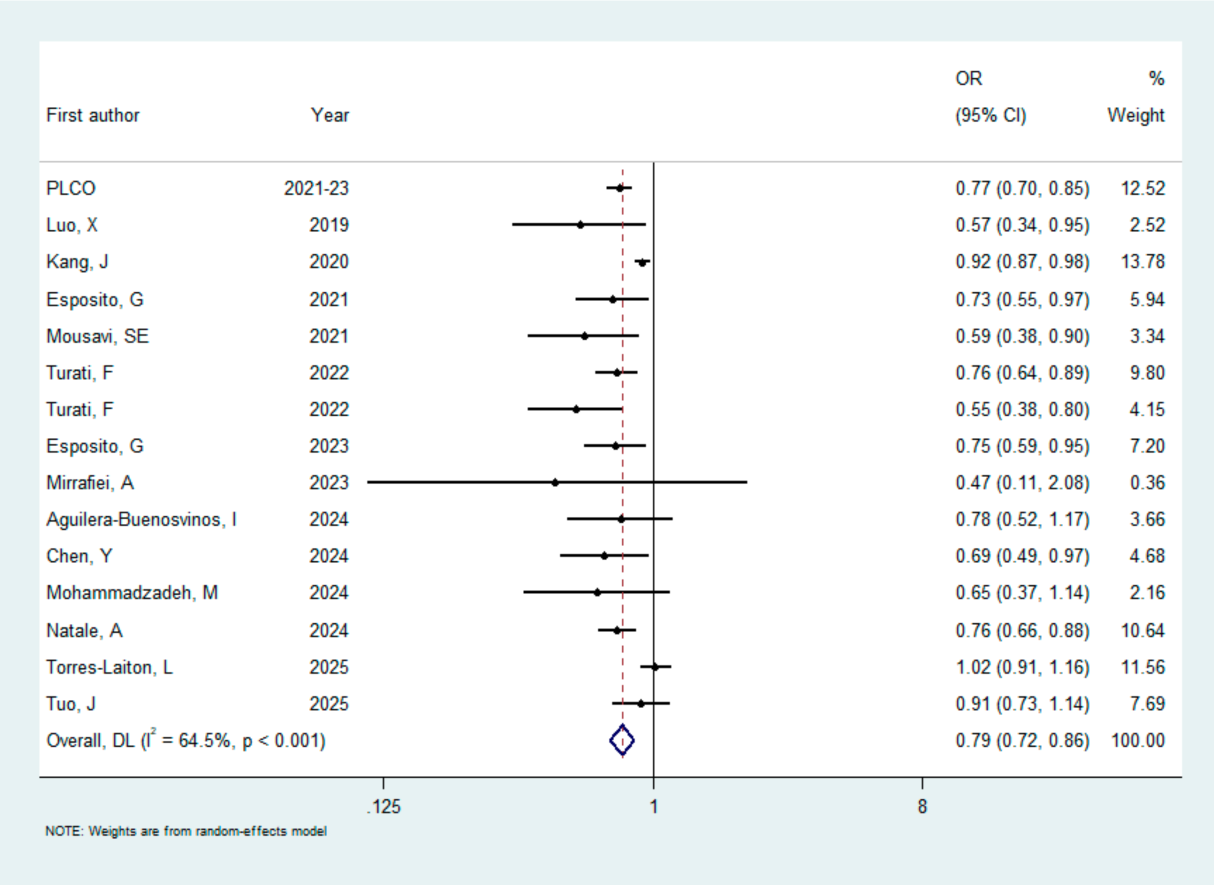

Figure S2. Pooled effect size of PLCO as a single study in combination with other studies

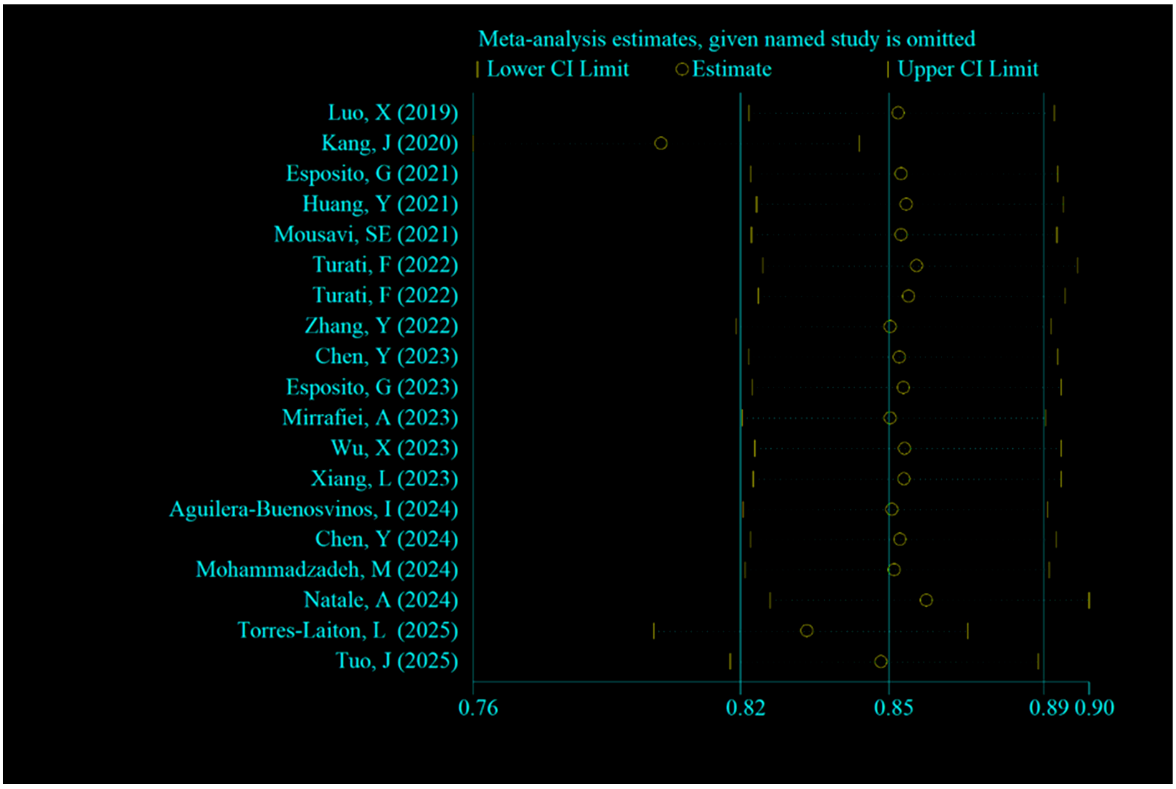

Figure S3. The sensitivity analysis of included studies
